# POPULATION ATTRIBUTABLE FRACTIONS FOR THE CONTRIBUTION OF HEARING LOSS ON DEMENTIA RISK ACROSS COHORT STUDIES

**DOI:** 10.1093/geroni/igad104.0765

**Published:** 2023-12-21

**Authors:** Jason Smith, Emily Burg, A Richey Sharrett, Nicholas Reed, Frank Lin, Josef Coresh, Jennifer Deal, Emily Ishak

**Affiliations:** Johns Hopkins Bloomberg School of Public Health, Baltimore, Maryland, United States; University of Wisconsin-Madison, Madison, Wisconsin, United States; Johns Hopkins Bloomberg School of Public Health, Baltimore, Maryland, United States; Johns Hopkins Bloomberg School of Public Health, Baltimore, Maryland, United States; Johns Hopkins, Baltimore, Maryland, United States; Johns Hopkins University, Baltimore, Maryland, United States; Johns Hopkins University, Baltimore, Maryland, United States; Columbia University Irving Medical Center, New York City, New York, United States

## Abstract

Estimates range from 2-8% of dementia cases in the US are attributable to hearing loss. Here, we investigate measurement considerations in quantifying the population attributable fraction (PAF) of dementia from hearing loss to highlight how differences in methodology can underestimate the PAF. We use two cohort studies of older adults in the US: the National Health and Aging Trends Study (NHATS), a nationally-representative sample of older Medicare beneficiaries; and the Atherosclerosis Risk in Communities (ARIC) Study, a large, community-based cohort study with longitudinal follow-up. Each study collects reference-standard hearing measures on participants. We have two specific aims. First, we compare two valid, formula-based approaches to quantifying the PAF. The advantages of quantifying a PAF using a well-characterized, biracial, community-based sample with a longitudinal design are weighed against those of using a nationally-representative sample with a cross-sectional design (moderate or greater hearing loss: PAF in NHATS, 16.9% [95% CI: 4.1-28.7%]; PAF in ARIC, 13.2% [95% CI: 2.2-20.9%]), highlighting the need for longitudinal designs in studies of the PAF from hearing loss. Second, we describe the impact of subjective versus objective measures of hearing loss on the PAF. In ARIC, subjective hearing loss was not associated with incident dementia (hazard ratio <1.0). PAFs of dementia from objective hearing loss were 6- to 8-fold greater than PAFs previously quantified using self-reported hearing measures in population-based studies. Our findings have broad implications for the inclusion of hearing loss as a potentially modifiable risk factor in clinical and public health initiative for dementia prevention.

